# Supplementary material for: Murine MPDZ‐linked hydrocephalus is caused by hyperpermeability of the choroid plexus
Source: EMBO Mol Med. 2018 Dec 5;11(1):e9540. doi: 10.15252/emmm.201809540 (PMC6328942; doi:10.15252/emmm.201809540)
Supplement: Supplementary file 1 — Expanded View Figures PDF [file EMMM-11-e9540-s001.pdf]

Expanded View Figures

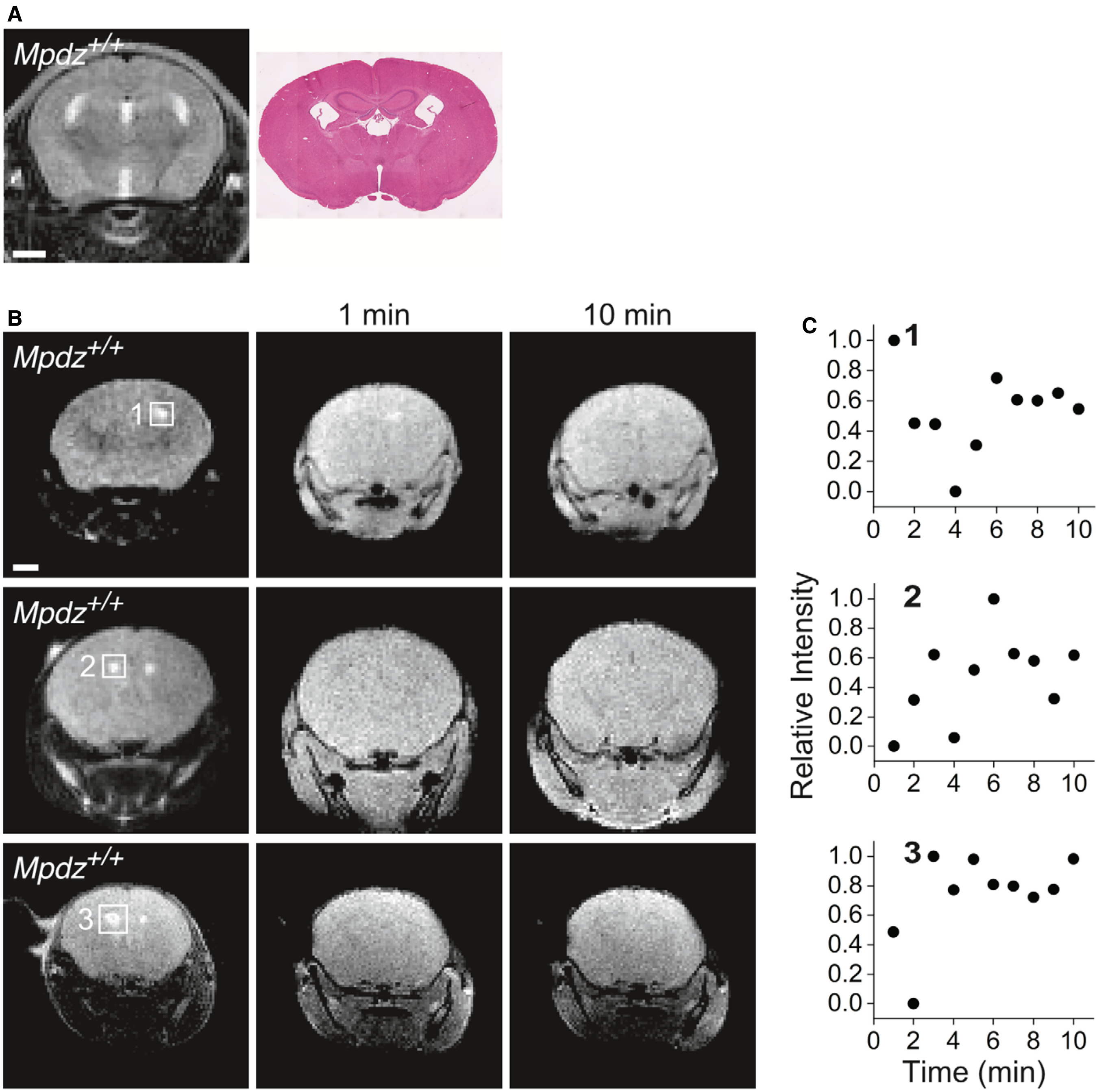

Figure EV1. No contrast medium is detected in the brain ventricles of *Mpdz*$^{+/+}$ mice.

A Coronal T2-weighted MR image and an anatomically corresponding HE-stained section. Scale bar, 1 mm.

B Triplicate rows of T2-weighted and T1-weighted coronal images 1 and 10 min post-contrast medium injection. The squares surround the locations of the lateral ventricles. Each row corresponds to one P18-P21 mouse. Scale bar, 1 mm.

C Time courses of the normalized T1-weighted image intensities corresponding to the locations of the areas surrounded by numbered squares in the T2-weighted images.

© 2018 The Authors

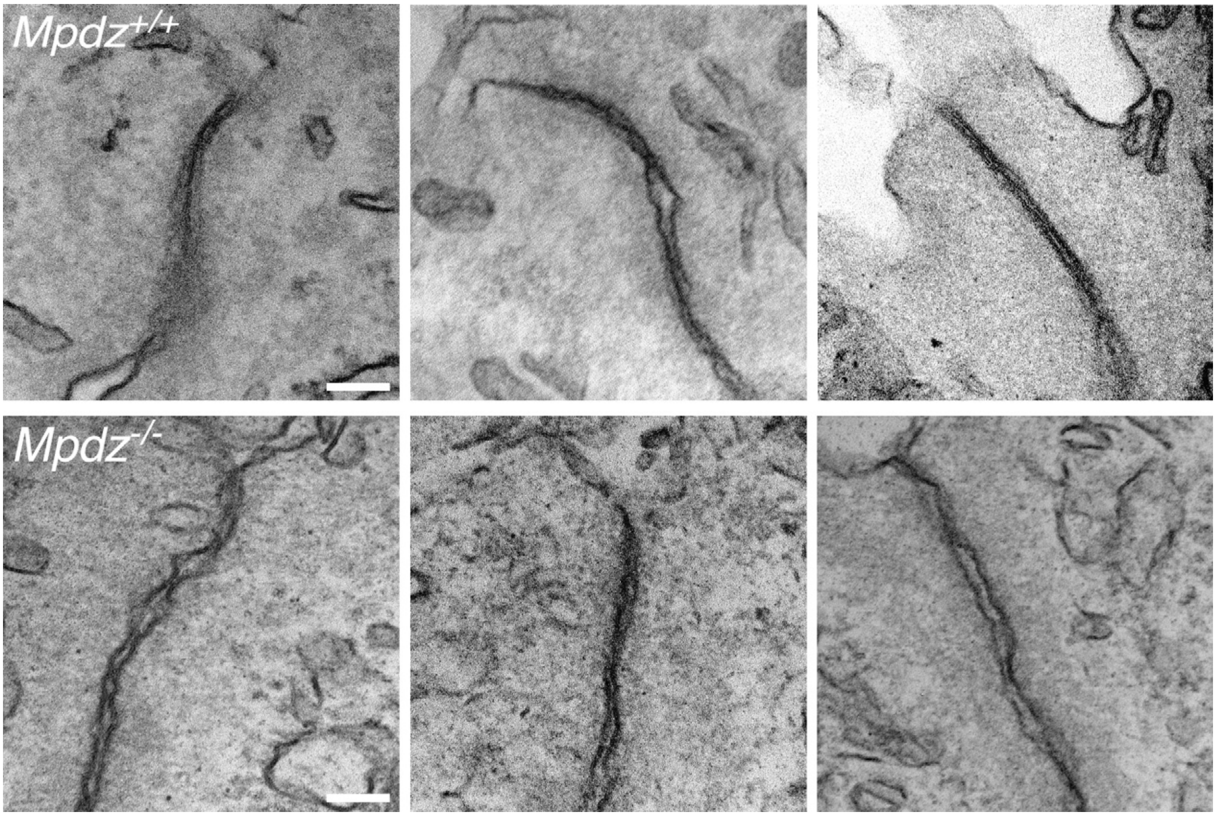

Figure EV2. The structure of CPEC tight junctions of P12-14 *Mpdz*$^{-/-}$ mice is defective.

A gallery of three TEM images of tight junctions between the CPECs from lateral ventricle CP villi of *Mpdz*$^{+/+}$ and *Mpdz*$^{-/-}$ P12-14 mice. Scale bars, 100 nm.

© 2018 The Authors

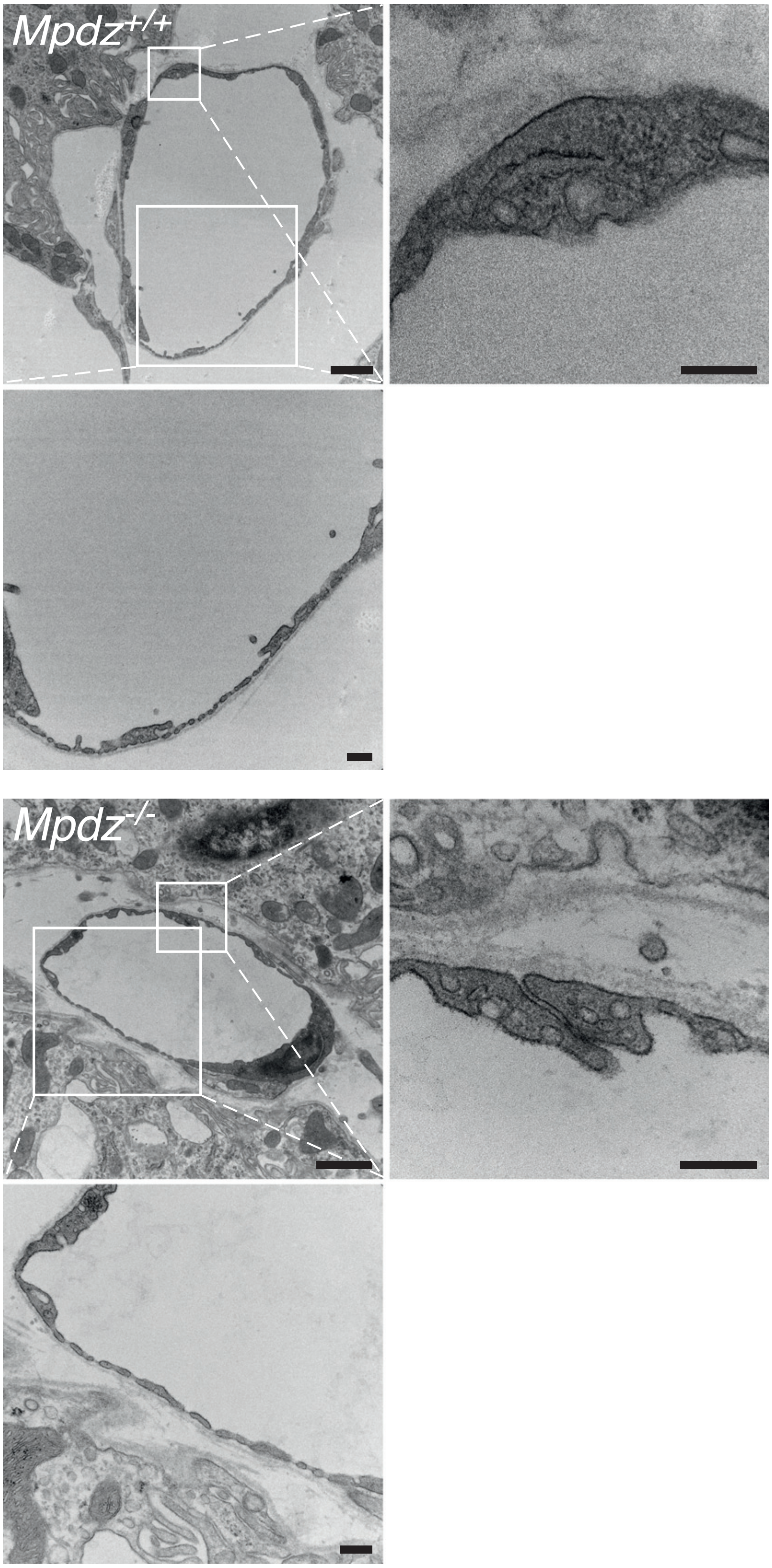

Figure EV3. Capillaries between CPECs of *Mpdz*$^{-/-}$ mice do not harbor structural defects.

TEM images of sections of capillaries from lateral ventricle CP villi of *Mpdz*$^{+/+}$ and *Mpdz*$^{-/-}$ P15-P21 mice. The magnified fields show intercellular junctions between endothelial cells or fenestrae. Scale bars, 1 μm; insets, 250 nm.

© 2018 The Authors
