# Supplementary figures and images for: Murine MPDZ‐linked hydrocephalus is caused by hyperpermeability of the choroid plexus
Source: EMBO Mol Med. 2018 Dec 5;11(1):e9540. doi: 10.15252/emmm.201809540 (PMC6328942; doi:10.15252/emmm.201809540)

Full-length immunoblots for Figure 4D

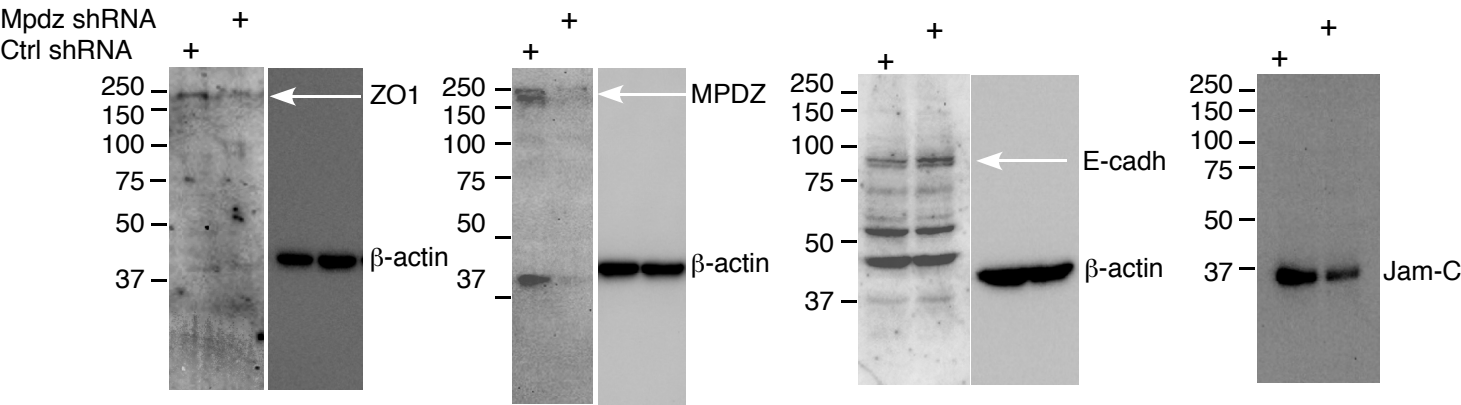

Supplement: Supplementary file 4 — Source Data for Figure 4 [file EMMM-11-e9540-s003.pdf]

Full-length immunoblots for Figure 5E

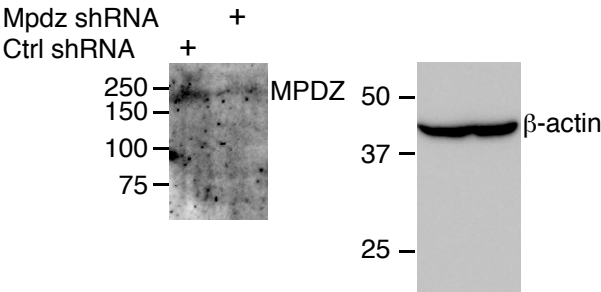

Supplement: Supplementary file 5 — Source Data for Figure 5 [file EMMM-11-e9540-s004.pdf]

# Full-length immunoblots for Figure 7C

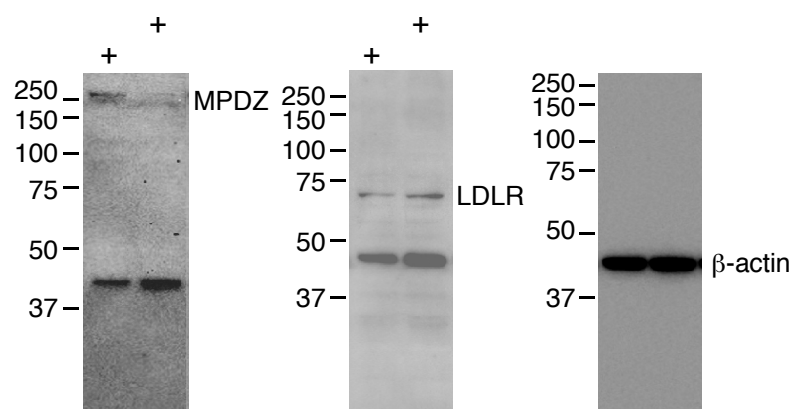

Supplement: Supplementary file 6 — Source Data for Figure 7 [file EMMM-11-e9540-s005.pdf]

## Full-length immunoblots for Figure 8E

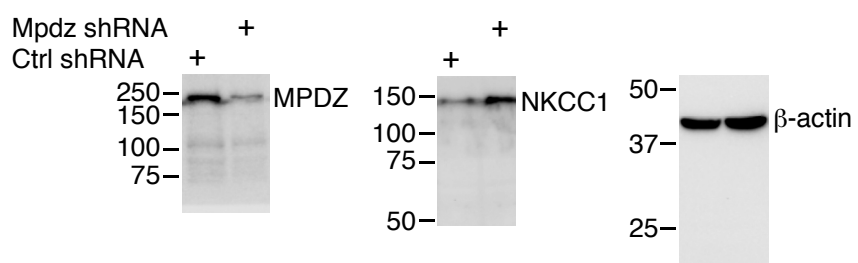

Supplement: Supplementary file 7 — Source Data for Figure 8 [file EMMM-11-e9540-s006.pdf]
